# Comparison of an HPLC-UV system (LM1010) and UPLC-MS/MS for plasma voriconazole measurement in routine clinical practice

**DOI:** 10.1186/s40780-026-00573-3

**Published:** 2026-04-17

**Authors:** Junichi Nakagawa, Kayo Ueno, Katsuyoshi Osanai, Masahiro Ishiyama, Miyuki Matsushita, Satoru Morikawa, Hirofumi Tomita, Takenori Niioka

**Affiliations:** 1 Department of Pharmacy, Hirosaki University Hospital, 53 Hon-cho, Hirosaki, Aomori, 036-8563 Japan; 2 Department of Pharmacy, Aomori Prefectural Central Hospital, Aomori, Japan; 3 Department of Clinical Laboratory, Hirosaki University Hospital, Aomori, Japan; 4 Department of Chromatography Sales, Hitachi High-Tech Analysis Corporation, Tokyo, Japan; 5 Department of Cardiology and Nephrology, Hirosaki University Graduate School of Medicine, Aomori, Japan; 6 Department of Pharmaceutical Science, Hirosaki University Graduate School of Medicine, Aomori, Japan

**Keywords:** Voriconazole, LM1010, Therapeutic drug monitoring, High-performance liquid chromatography with ultraviolet-visible detection

## Abstract

**Background:**

We aimed to evaluate the clinical utility of the LM1010 high-performance liquid chromatography with ultraviolet-visible detection instrument for measuring plasma concentrations of voriconazole (VRCZ) under routine clinical conditions, including in patients with diverse clinical backgrounds.

**Methods:**

In total, 213 samples were collected from two hospitals (groups A and B). The plasma samples were analyzed using LM1010 and ultra performance liquid chromatography (UPLC)-tandem mass spectrometry (MS/MS). Laboratory test values and information on concomitant medications were collected from electronic medical records.

**Results:**

In total, 66 samples from group A and 135 samples from group B were included in the analysis.　Passing–Bablok regression analysis showed that in group A (*n* = 66), the slope was 0.972 (95% confidence interval [CI], 0.929–1.000) and the intercept −0.024 (95% CI, −0.100–0.056), indicating no significant proportional or constant bias. In group B (*n* = 135), the slope was 0.956 (95% CI, 0.930–0.975) and the intercept −0.006 (95% CI, −0.053–0.025), indicating a slight proportional bias but no constant bias. The Bland–Altman analysis demonstrated proportional bias in both groups A and B, and the slopes of the regression lines were 0.045 (95% CI, 0.011–0.079) and 0.038 (95% CI, 0.025–0.052), respectively. Interfering peaks were observed in the chromatograms of eight samples in group B, but their effect on the intermethod difference was small. No clinical laboratory test values or concomitant medications were identified as factors affecting the intermethod difference.

**Conclusions:**

The measurement of VRCZ plasma concentration by LM1010 was robust under heterogeneous clinical conditions and was useful for clinical therapeutic drug monitoring of VRCZ.

**Supplementary Information:**

The online version contains supplementary material available at 10.1186/s40780-026-00573-3.

## Introduction

Voriconazole (VRCZ), an azole antifungal agent, is widely used for the treatment and prophylaxis of invasive fungal infections. The plasma concentration of VRCZ is related to its therapeutic effect and the risk of side effects, such as liver dysfunction; therefore, therapeutic drug monitoring (TDM) is recommended [1]. The current gold standard for measurement of the plasma concentration of VRCZ is liquid chromatography-tandem mass spectrometry (LC-MS/MS) [2]. However, due to the high cost of equipment and the need for skilled technicians, only a limited number of institutions in Japan are able to perform in-house testing, and most rely on external laboratories [3, 4].

VRCZ is primarily metabolized to voriconazole *N*-oxide (VNO) by cytochrome P450 (CYP) 2C19, with contributions from CYP2C9 and CYP3A4 [5, 6]. The pharmacokinetics of VRCZ are highly variable among individuals due to *CYP2C19* polymorphisms, and even within the same individual, they can vary significantly over several days in response to changes in the inflammatory response [7]. Therefore, optimal individualized VRCZ therapy requires frequent measurement of plasma concentrations and rapid feedback of the results; however, with outsourcing, it takes approximately 5 days from blood sampling to receive feedback from the results in Japan [4]. Thus, there is a clinical need for a cost-effective and user-friendly analytical platform that enables rapid in-hospital measurement of VRCZ plasma concentrations.

In Japan, the LM1010 high-performance liquid chromatography with ultraviolet-visible detection (HPLC-UV) system, which has been approved for clinical use as a medical device, is available for the measurement of plasma VRCZ concentrations. However, the robustness of this system under heterogeneous clinical and analytical conditions encountered in routine practice remains insufficiently characterized. In particular, the influence of differences in clinical indications, sample handling procedures, measurement timing, and laboratory environments on intermethod agreement has not been fully examined. In real-world settings, VRCZ is used both for prophylaxis and treatment across diverse patient populations, and plasma samples may be processed under varying pre-analytical conditions, including delayed analysis after frozen storage or immediate same-day measurement.

The purpose of this study was to evaluate the clinical utility and analytical accuracy of the LM1010 instrument for measuring plasma VRCZ concentrations under real-world clinical conditions. By comparing LM1010 with ultra performance LC (UPLC)-MS/MS across two independent clinical settings involving different therapeutic indications and sample handling conditions and by systematically assessing the impact of concomitant medications and laboratory test values on intermethod variability, we aimed to clarify the robustness of the LM1010 for routine TDM.

## Methods

### Chemical and reagents

The VRCZ used in the UPLC-MS/MS analyses was purchased from LKT Laboratories (St. Paul, MN, USA). VRCZ-d3 was purchased from Cayman Chemical (Ann Arbor, MI, USA) and used as an internal standard for UPLC-MS/MS. The 5 µg/mL VRCZ standard solution and mobile phases A and B used in the LM1010 analysis were purchased from Hitachi High-Tech Analysis (Tokyo, Japan). The solid-phase extraction spin column set used for sample preparation for LM1010 analysis was also purchased from Hitachi High-Tech Analysis (Tokyo, Japan).

### Patients and blood sampling

Blood samples were collected from Japanese patients undergoing treatment with VRCZ at Aomori Prefectural Central Hospital between July 2019 and August 2021 (group A) or at Hirosaki University Hospital between June 2023 and December 2024 (group B). In group A, VRCZ was primarily used for the prophylaxis of infections in patients undergoing bone marrow transplantation, whereas in group B, it was mainly used for the treatment of invasive fungal infections. In both groups, blood samples were collected immediately before the next dose in heparin sodium tubes and centrifuged at 3,500 rpm for 10 min at 4 °C.

To ensure consistency and minimize interoperator variability, all plasma VRCZ concentration measurements were performed at Hirosaki University Hospital by a single experienced analyst using two different analytical platforms: the LM1010 HPLC-UV system and the UPLC-MS/MS system.

Laboratory test values on the same day as blood sampling for both groups A and B and information on concomitant medications administered from the day before to the day of blood sampling for group B were collected from electronic medical records. Because this study included retrospectively collected samples, the availability of chromatographic data differed between the two groups, and chromatograms were not available for retrospective evaluation in group A. In addition, detailed information on concomitant medications administered from the day before to the day of blood sampling was available only for group B. The study protocol was approved by the Ethics Committee of Hirosaki University Graduate School of Medicine (project identification code: 2023–036-1).

### Measurement of VRCZ plasma concentrations by LM1010

The plasma VRCZ concentration was measured using an automated HPLC-UV system (LM1010; Hitachi High-Tech Analysis, Tokyo, Japan). The plasma concentration of VRCZ was measured using LM1010 according to the operating instructions provided by Hitachi High-Tech. Separated plasma samples from group A were shipped frozen to Hirosaki University Hospital on the day of blood sampling and analyzed within 7 days of blood sampling. In group B, separated plasma samples were analyzed on the same day as blood collection. The plasma sample (150 µL) was loaded into a spin column preconditioned with 500 µL pretreatment solution A and 500 µL pretreatment solution B, and the column was centrifuged at 2,400 × *g* for 3 min at room temperature. After the column was washed with pretreatment solution B, the sample was eluted with 150 µL pretreatment solution C. The processed VRCZ sample was vortexed for 10 s and subjected to LM1010. HPLC conditions were the default settings, and peak selection for VRCZ as well as calculation of plasma concentrations were performed automatically by the analysis software of the LM1010 system.

### Measurement of VRCZ plasma concentrations by UPLC-MS/MS

The remaining aliquots for UPLC-MS/MS were stored at − 80 °C, and all measurements were performed within 6 months of sample collection to minimize potential degradation of analytes [8]. The UPLC-MS/MS system consisted of an ACQUITY UPLC System (Waters, MA, USA) and Xevo TQD (Waters). The conditions for plasma concentration analysis of VRCZ were described in our previous report [9]. The range of the VRCZ calibration curve was 0.25–10 µg/mL.

### Statistical procedures

Comparisons of VRCZ concentrations measured by LM1010 and clinical laboratory values between groups A and B were performed using the Mann-Whitney U test. Passing–Bablok regression analysis and Bland–Altman plots were performed to compare the plasma concentrations of VRCZ measured by LM1010 HPLC and UPLC-MS/MS. In the Bland–Altman plots, the limits of the agreement (LOAs) of intermethod differences were defined as the mean bias ±1.96 × standard deviation. The constant bias and proportional bias between LM1010 and UPLC-MS/MS were assessed in the Bland–Altman plots using one-sample t-tests and linear regression analysis, respectively. Patient samples with measured VRCZ plasma concentrations less than 0.2 µg/mL by LM1010 or less than 0.25 µg/mL by UPLC-MS/MS were excluded from the analyses because of the lower limit of quantification. Relationships between the day of blood sampling, clinical test values, and differences in measurement results between the two methods were assessed using Spearman’s rank correlation.

A *P* value less than 0.05 was considered to indicate statistical significance. Passing–Bablok regression analysis was performed using Validation-Support/Excel Ver.7.1 developed by the Japan Society of Clinical Chemistry [10]. Other statistical analyses were performed with SPSS 28.0 for Windows (SPSS IBM Japan Inc., Tokyo, Japan).

## Results

### Patient characteristics

In total, 73 samples were collected from 50 patients in group A, whereas in group B, 140 samples were collected from 32 patients. Blood sampling and LM1010 analysis were performed on 192 different days (69 days for group A and 123 days for group B). In group A, seven samples showed concentrations less than or equal to 0.2 µg/mL by LM1010; among these, six had values less than 0.25 mg/mL by UPLC-MS/MS, while one sample had a concentration of 0.4 µg/mL by UPLC-MS/MS. In group B, all five samples with VRCZ concentrations less than or equal to 0.2 µg/mL by LM1010 also had concentrations less than 0.25 µg/mL by UPLC-MS/MS in group B. In total, 12 samples (7 from group A and 5 from group B) with VRCZ concentrations below the lower limit of quantification were excluded from subsequent analyses. The laboratory test values for groups A and B are shown in Table 1. The median trough VRCZ concentration measured by LM1010 tended to be higher in group B than in group A (2.3 versus 1.9 µg/mL, respectively, *p* = 0.080). The median values of aspartate aminotransferase (AST), serum total bilirubin (T-Bil), and C-reactive protein (CRP) were significantly higher in group B than in group A (AST: 29 versus 24 U/L, respectively, *p* = 0.028; total bilirubin: 0.40 versus 0.36 mg/dL, respectively, *p* = 0.018; CRP: 1.22 versus 0.68 mg/dL, respectively, *p* = 0.026). The list of concomitant medications available for group B is shown in Supplementary Table 1. In total, 108 medications were administered between the day before and the day of blood sampling.

**Table 1:** Characteristics of laboratory test values on the same day as blood sampling

Explanatory variables	Group A			Group B			*P* value
	n	Median	Range	n	Median	Range	
Voriconazole by LM1010 (µg/mL)	66	1.9	(0.3 - 8.0)	135	2.3	(0.4 - 13.6)	0.080
Albumin (g/dL)	63	3.5	(1.2 - 4.4)	117	3.5	(1.1 - 5.1)	0.928
Serum creatinine (mg/dL)	63	0.61	(0.33 - 2.03)	117	0.73	(0.12 - 5.94)	0.228
Aspartate aminotransferase (U/L)	63	24	(10–143)	119	29	(8–264)	0.028
Alanine aminotransferase (U/L)	63	18	(6–334)	119	22	(5–134)	0.287
Serum total bilirubin (mg/dL)	63	0.36	(0.14 - 8.72)	113	0.40	(0.20 - 19.00)	0.018
C-reative protein (mg/dL)	65	0.68	(0.01 - 21.970	122	1.22	(0.02 - 39.87)	0.026

### Chromatograms by LM1010

In the LM1010 analysis, interfering peaks partially overlapping with the VRCZ peak were detected in eight patient samples from group B (Supplementary Fig. 1). There was no single concomitant medication that was administered to every patient with interfering peaks. Although markedly elevated bilirubin levels were observed in two samples with interfering peaks, no consistent abnormalities in laboratory test values were identified across the eight samples (Supplementary Table 2). In addition, similar peak interference was not consistently observed in samples with elevated T-Bil levels across the entire study population, and no systematic association could be established.

### Comparison of LM1010 and UPLC-MS/MS

**Fig. 1:**
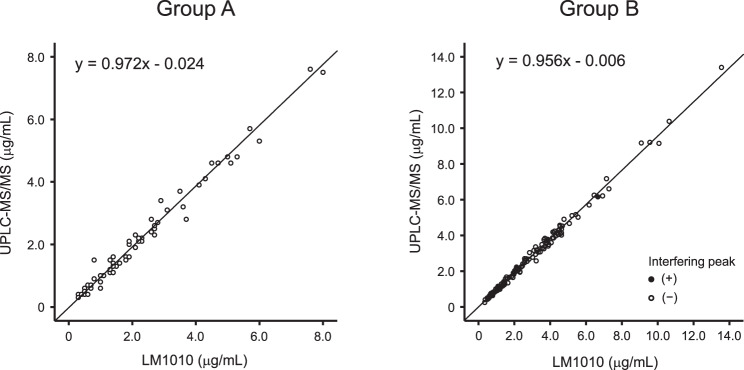
Relationships between voriconazole plasma concentrations measured by LM1010 and UPLC-MS/MS

Passing–Bablok regression analysis showed that in group A (*n* = 66), the slope was 0.972 (95% confidence interval [CI], 0.929–1.000) and the intercept −0.024 (95% CI, −0.100–0.056), indicating no significant proportional or constant bias. In group B (*n* = 135), the slope was 0.956 (95% CI, 0.930–0.975) and the intercept −0.006 (95% CI, −0.053–0.025), indicating a slight proportional bias but no constant bias (Fig. 1).

**Fig. 2:**
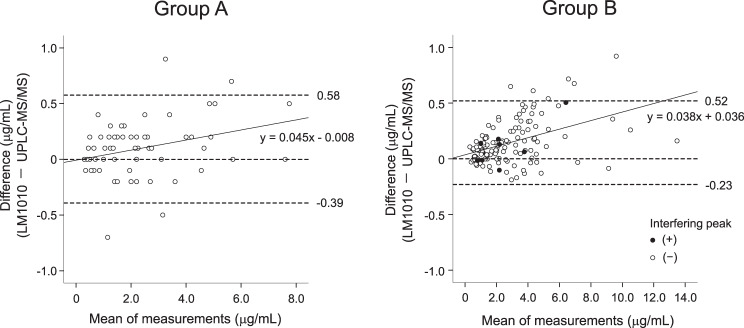
Bland–Altman plots of differences between LM1010 and UPLC-MS/MS measurements of VRCZ. Mean of measurements represents the mean voriconazole concentration measured by LM1010 and UPLC-MS/MS. The dashed lines indicate the mean difference ±1.96 × standard deviation and zero, and the solid line represents the regression line

Bland–Altman plots of the differences and ratios between the two methods are shown in Fig. 2 and Supplementary Fig. 2. In group A, the mean intermethod difference in VRCZ plasma concentration was 0.09 µg/mL (95% CI, 0.03–0.15), with constant bias (*p* = 0.003). For all but four (6.2%) samples, the differences were within the LOA (−0.39 to 0.58 µg/mL). Linear regression analysis showed that the slope of the regression line was 0.045 (95% CI, 0.011–0.079), and significant proportional bias was observed (*r*^2^ = 0.098, *p* = 0.010). In group B, the mean intermethod difference was 0.14 µg/mL (95% CI, 0.11–0.18), with constant bias (*p* < 0.001). For all but six (4.4%) samples, the differences were within the LOA (−0.23 to 0.52 µg/mL). Linear regression analysis showed that the slope of the regression line was 0.038 (95% CI, 0.025–0.052), and significant proportional bias was observed (*r*^2^ = 0.194, *p* < 0.001). The difference for all eight samples in which interference peaks were detected was within the LOA (Supplementary Table 2). The ratio-based Bland–Altman plots complement the difference-based plots by assessing the stability of relative agreement across the concentration range. In the ratio plot, the mean ratios of the measured VRCZ plasma concentration by LM1010 to UPLC-MS/MS were 1.055 (95% CI, 1.013–1.096, *p* < 0.001) in group A and 1.061 (95% CI, 1.048–1.074, *p* < 0.001) in group B, indicating a small, constant bias toward higher concentrations measured by LM1010. No significant proportional bias was found between the ratio and means of measurements in both groups A and B (*p* = 0.746 and 0.225, respectively).

Table 2 shows the agreement between LM1010 and UPLC-MS/MS based on concentration category classifications according to the recommended therapeutic range for VRCZ in Japan [11]. Intermethod discordance classifications were observed in four samples (6.1%)　in group A and 10 samples (7.4%) in group B. A list of samples showing discordant classifications is provided in Supplementary Table 3; in all but one of these samples, the VRCZ plasma concentrations measured by LM1010 were higher than those measured by UPLC-MS/MS. There was no significant association between intermethod differences in VRCZ concentrations measured by LM1010 and UPLC-MS/MS and the time elapsed since the initiation of TDM in either group A or group B (*p* = 0.303 and 0.102, respectively).

**Table 2:** Agreement between LM1010 and UPLC-MS/MS measurements of voriconazole plasma concentrations according to recommendations in Japan

		UPLC-MS/MS		
LM1010	**Group A**	Low (<1 µg/mL)	Therapeutic (1–4 µg/mL)	High (>4 µg/mL)
	Low (<1 µg/mL)	14	1	0
	Therapeutic (1–4 µg/mL)	2	38	0
	High (>4 µg/mL)	0	1	10
	**Group B**	Low (<1 µg/mL)	Therapeutic (1–4 µg/mL)	High (>4 µg/mL)
	Low (<1 µg/mL)	21	0	0
	Therapeutic (1–4 µg/mL)	4	77	0
	High (>4 µg/mL)	0	6	27

## Discussion

In this study, we compared VRCZ plasma concentrations measured by LM1010 and UPLC-MS/MS in patients with a variety of concomitant medications based on samples collected from two institutions. Despite heterogeneity in clinical indications, concomitant medications, and samples, plasma VRCZ concentrations measured by LM1010 showed strong agreement with those measured by UPLC-MS/MS. These findings indicated that LM1010 provided sufficiently robust and clinically reliable measurements for routine VRCZ TDM.

Our results showed that the measured VRCZ plasma concentrations were approximately 6% higher by LM1010 than by UPLC-MS/MS in both groups, indicating a small but significant systematic bias between the two methods. For the recommended therapeutic plasma concentration range for VRCZ in Japan (1–4 µg/mL) [11], the systematic bias observed in this study corresponds to an absolute difference of approximately 0.06–0.24 µg/mL. This tendency toward higher values by LM1010 may be attributable to the inherent characteristics of UV-based detection, whereby at higher analyte concentrations, enlargement of the VRCZ peak increases the relative contribution of peak tailing and minor co-eluting components to the integrated peak area, resulting in slight overestimation. Passing–Bablok regression analysis further supported the overall agreement between the two methods. No constant or proportional bias was observed in group A, whereas group B showed slight proportional bias without constant bias. These findings are consistent with the difference-based Bland–Altman plots, in which absolute intermethod differences increased slightly as the VRCZ plasma concentration increased. However, no proportional bias was observed in the ratio-based Bland–Altman plots, indicating that the relative agreement between the LM1010 and UPLC-MS/MS results remained stable across the concentration range. Furthermore, the LOA values in the difference-based plots were −0.38 to 0.58 µg/mL in group A and −0.23 to 0.52 µg/mL in group B, which were small compared with the recommended therapeutic range in Japan [11]. Discordant concentration category classifications based on the recommended therapeutic range were observed in fewer than 8% of samples in each group, and only one sample per group showed both an intermethod difference outside the limits of agreement and a discordant classification. These results suggest that the observed inter-method differences are unlikely to meaningfully affect clinical decision-making in routine VRCZ TDM. In addition, no significant association was observed between the day of blood sampling and the intermethod difference over extended sampling periods, supporting the long-term robustness of LM1010 measurements. While recent clinical studies have highlighted the practical advantages of LM1010 in VRCZ TDM in terms of workflow efficiency and turnaround time [12, 13], our current findings extend these observations by demonstrating the analytical robustness of LM1010 across heterogeneous clinical conditions.

In this study, CRP, AST, and total bilirubin levels were significantly higher in group B than in group A, indicating distinct clinical backgrounds between the two cohorts (Table 1). In addition, although the difference did not reach statistical significance, the median trough VRCZ concentration measured by LM1010 tended to be higher in group B than in group A. Inflammatory conditions are known to suppress the expression of pregnane X receptor, thereby reducing CYP2C19 expression and increasing VRCZ exposure [9, 14]. Moreover, impaired hepatic function has also been associated with decreased voriconazole metabolism and elevated plasma concentrations [15]. Therefore, the trend toward higher measured VRCZ concentrations in group B may be explained, at least in part, by increased inflammatory activity, as reflected by elevated CRP levels, and mildly impaired liver function. These findings reflect clinically relevant background differences rather than limitations in the analytical performance of the LM1010. Importantly, despite variations in patient backgrounds and VRCZ exposure levels between institutions, LM1010 consistently provided accurate measurements that reflected these clinical differences.

There were several limitations to this study. First, the cause of the interfering peaks detected in the LM1010 analysis was not identified. Although trimethoprim–sulfamethoxazole had been administered to most patients with interfering peaks, it was not common among all such samples. We evaluated a mixed standard solution of trimethoprim and sulfamethoxazole using LM1010 analysis; however, no peaks corresponding to the interfering peaks were detected (data not shown). Second, the interval between administration of concomitant medications and blood sampling was not evaluated, and medication adherence on the day before blood sampling was also not assessed. Concomitant medications with short elimination half-lives may not have been present in the plasma at the time of blood collection.

In conclusion, plasma VRCZ concentrations measured using the LM1010 showed strong agreement with those measured by UPLC-MS/MS under routine clinical conditions. These findings support that measurement of VRCZ plasma concentration using LM1010 is robust in patients with diverse backgrounds and is useful for clinical TDM of VRCZ.

## Electronic supplementary material

Below is the link to the electronic supplementary material.

Supplementary material 1

## Data Availability

The datasets used and/or analyzed during the current study are available from the corresponding author on reasonable request.

## Abbreviations

AST: Aspartate aminotransferase
CRP: C-reactive protein
HPLC-UV: High-performance liquid chromatography with ultraviolet-visible detection
T-Bil: Total bilirubin
TDM: Therapeutic drug monitoring
UPLC-MS/MS: Ultra performance liquid chromatography-tandem mass spectrometry
VRCZ: Voriconazole

## References

[1] Takesue Y, Hanai Y, Oda K, Hamada Y, Ueda T, Mayumi T, et al. Clinical practice guideline for the therapeutic drug monitoring of voriconazole in non-Asian and Asian adult patients: consensus review by the Japanese Society of Chemotherapy and the Japanese Society of therapeutic drug monitoring. Clin Ther. 2022;44(12):1604–23. 10.1016/j.clinthera.2022.10.005. 36424314 10.1016/j.clinthera.2022.10.005

[2] Mak J, Sujishi KK, French D. Development and validation of a liquid chromatography–tandem mass spectrometry (LC–MS/MS) assay to quantify serum voriconazole. J Chromatogr B. 2015;986-987:94–99. 10.1016/j.jchromb.2015.02.011. 10.1016/j.jchromb.2015.02.011 25725319

[3] Hamada Y, Tokimatsu I, Mikamo H, Kimura M, Seki M, Takakura S, et al. Practice guidelines for therapeutic drug monitoring of voriconazole: a consensus review of the Japanese Society of Chemotherapy and the Japanese Society of therapeutic drug monitoring. J Infect Chemother. 2013;19(3):381–92. 10.1007/s10156-013-0607-8. 23673473 10.1007/s10156-013-0607-8 PMC3682092

[4] Yasu T, Nomura Y, Gando Y, Matsumoto Y, Sugita T, Kosugi N, et al. High-performance liquid chromatography for ultra-simple determination of plasma voriconazole concentration. JoF. 2022;8(10):1035. 10.3390/jof8101035. 36294600 10.3390/jof8101035 PMC9604553

[5] Theuretzbacher U, Ihle F, Derendorf H. Pharmacokinetic/pharmacodynamic profile of voriconazole. Clin Pharmacokinet. 2006;45(7):649–63. 10.2165/00003088-200645070-00002. 16802848 10.2165/00003088-200645070-00002

[6] Hyland R, Jones BC, Smith DA. Identification of the cytochrome P450 enzymes involved in the N-oxidation of voriconazole. Drug Metab Dispos. 2003;31(5):540–47. 10.1124/dmd.31.5.540. 12695341 10.1124/dmd.31.5.540

[7] Klomp SD, Veringa A, Alffenaar J-WC, de Boer MGJ, Span LFR, Guchelaar HJ, et al. Inflammation altered correlation between CYP2C19 genotype and CYP2C19 activity in patients receiving voriconazole. Clin Transl Sci. 2024;17(7):e13887. 10.1111/cts.13887. 10.1111/cts.13887 PMC11250525 39010708

[8] Gordien JB, Pigneux A, Vigouroux S, Tabrizi R, Accoceberry I, Bernadou JM, et al. Simultaneous determination of five systemic azoles in plasma by high-performance liquid chromatography with ultraviolet detection. J Pharmaceut Biomed. 2009;50(5):932–38. 10.1016/j.jpba.2009.06.030. 10.1016/j.jpba.2009.06.030 19608374

[9] Aiuchi N, Nakagawa J, Sakuraba H, Takahata T, Kamata K, Saito N, et al. Impact of polymorphisms of pharmacokinetics-related genes and the inflammatory response on the metabolism of voriconazole. Pharmacol Res & Perspec. 2022;10(2):e00935. 10.1002/prp2.935. 10.1002/prp2.935 PMC8866912 35199485

[10] Japan Society of Clinical Chemistry. 18 May 2026. https://jscc-jp.gr.jp/?page_id=1145.

[11] Kato H, Umemura T, Hagihara M, Shiota A, Asai N, Hamada Y, et al. Development of a therapeutic drug-monitoring algorithm for outpatients receiving voriconazole: a multicentre retrospective study. Brit J Clin Pharma. 2024;90(5):1222–30. 10.1111/bcp.16004. 10.1111/bcp.16004 38320604

[12] Oda K, Uchino S, Kurogi K, Horikawa M, Matsumoto N, Yonemaru K, et al. Clinical evaluation of an authorized medical equipment based on high performance liquid chromatography for measurement of serum voriconazole concentration. J Pharm Health Care Sci. 2021;7(1):42. 10.1186/s40780-021-00225-8. 34749825 10.1186/s40780-021-00225-8 PMC8576885

[13] Morikawa S, Yagi Y, Okazaki M, Yanagisawa N, Ishida T, Jobu K, et al. Rapid therapeutic drug monitoring of voriconazole based on high-performance liquid chromatography: a single-center pilot study in outpatients. Antibiot (Basel). 2025;14(5):474. 10.3390/antibiotics14050474. 10.3390/antibiotics14050474 PMC12108353 40426540

[14] van Wanrooy MJP, Span LFR, Rodgers MGG, van den Heuvel ER, Uges DRA, van der Werf TS, et al. Inflammation is associated with voriconazole trough concentrations. Antimicrob Agents Chemother. 2014;58(12):7098–101. 10.1128/aac.03820-14. 25223994 10.1128/AAC.03820-14 PMC4249508

[15] Hu L, Su Y, Tang X, Li Y, Feng J, He G. Therapeutic drug monitoring and safety of voriconazole in patients with liver dysfunction. Antimicrob Agents Chemother. 2024;68(11):e0112624. 10.1128/aac.01126-24. 10.1128/aac.01126-24 PMC11539214 39431818

