# Supplementary material for: Comparison of an HPLC-UV system (LM1010) and UPLC-MS/MS for plasma voriconazole measurement in routine clinical practice
Source: J Pharm Health Care Sci. 2026 Apr 17;12:54. doi: 10.1186/s40780-026-00573-3 (PMC13214191; doi:10.1186/s40780-026-00573-3)
Supplement: Supplementary file 1 — Supplementary material 1 [file 40780_2026_573_MOESM1_ESM.docx]

*Journal of Pharmaceutical Health Care and Sciences*

**Comparison of an HPLC-UV system (LM1010) and UPLC-MS/MS for plasma voriconazole measurement in routine clinical practice**

Junichi Nakagawa^1^, Kayo Ueno^1^, Katsuyoshi Osanai^2^, Masahiro Ishiyama^3^, Miyuki Matsushita^4^, Satoru Morikawa^4^, Hirofumi Tomita^3, 5^, Takenori Niioka^1, 6^

^1^Department of Pharmacy, Hirosaki University Hospital, Aomori, Japan

^2^Department of Pharmacy, Aomori Prefectural Central Hospital, Aomori, Japan

^3^Department of Clinical Laboratory, Hirosaki University Hospital, Aomori, Japan and ^4^ Department of Chromatography Sales, Hitachi High-Tech Analysis Corporation, Tokyo, Japan

^5^Department of Cardiology and Nephrology, Hirosaki University Graduate School of Medicine

^6^Department of Pharmaceutical Science, Hirosaki University Graduate School of Medicine, Aomori, Japan

Correspondence

Takenori Niioka, PhD, Department of Pharmacy, Hirosaki University Hospital, 53 Hon-cho, Hirosaki, Aomori 036-8563, Japan

E-mail: t-niioka@hirosaki-u.ac.jp

ORCID: 0000-0002-4560-6423


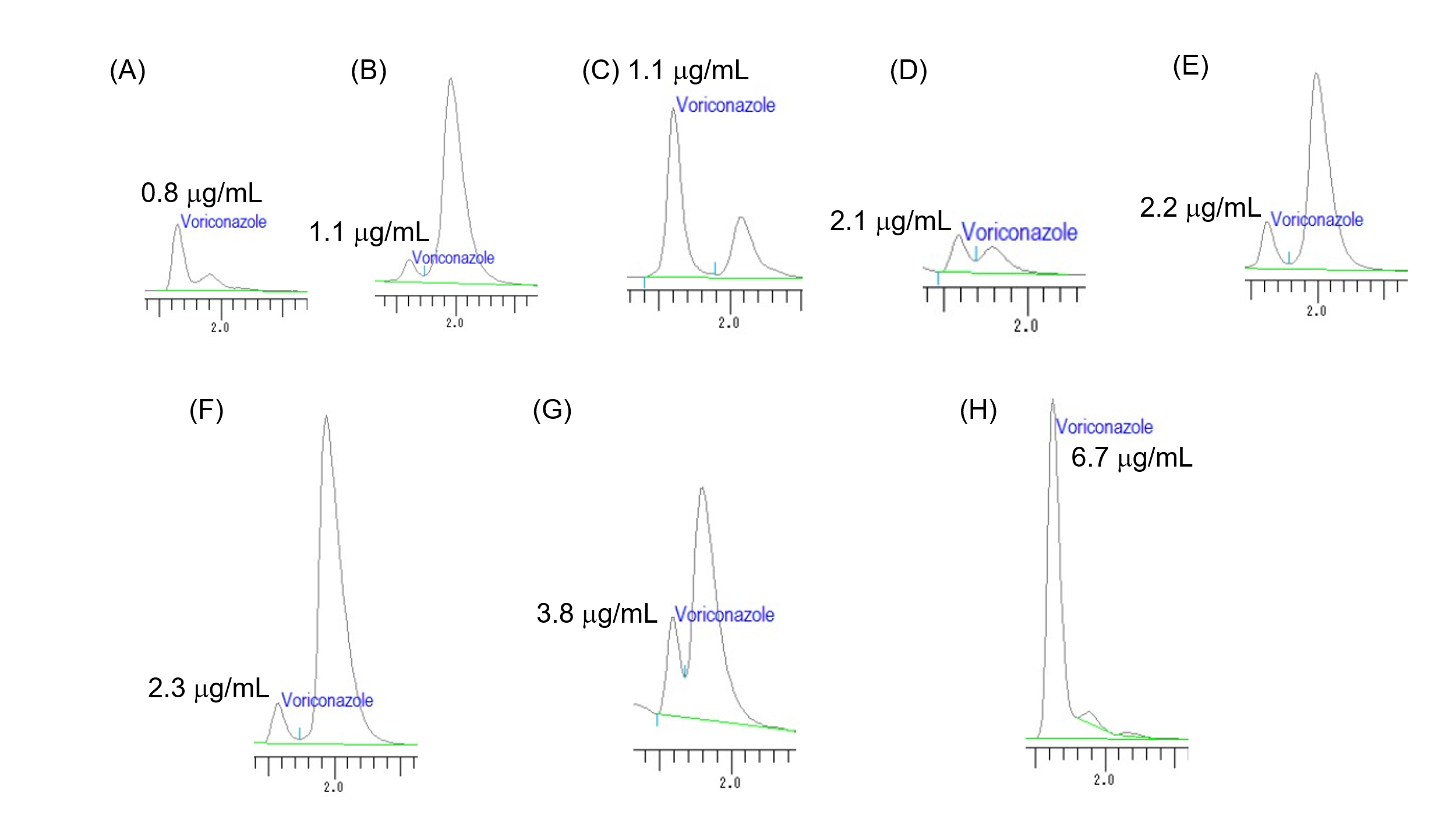
**Supplementary Fig. 1 Chromatograms obtained by LM1010 showing interfering peaks partially overlapping with the voriconazole peak in eight patient samples from group B.**

The numbers in each panel indicate the voriconazole plasma concentrations measured by LM1010.


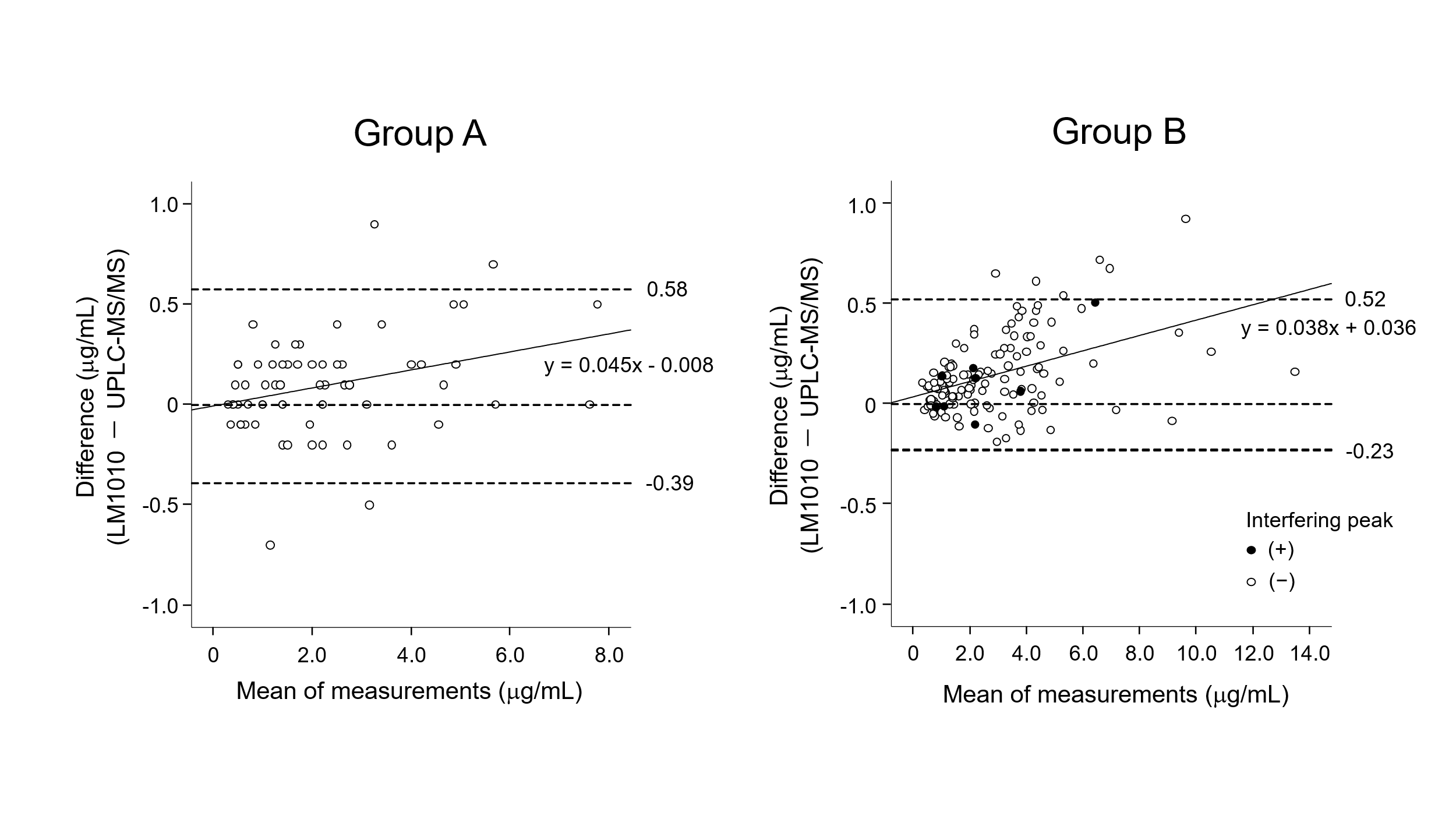


**Supplementary Fig. 2 Bland-Altman plots of ratios between LM1010 and UPLC-MS/MS measurements of VRCZ.**

Mean of measurements represents the mean voriconazole concentration measured by LM1010 and UPLC-MS/MS. The dashed lines indicate the mean difference ± 1.96 × standard deviation and 1, and the solid line represents the regression line.

**Supplementary Table 1 List of concomitant drugs in group B.**

**Supplementary Table 2 Characteristics of patient samples in group B in which the interfering peak was detected by LM1010.**

**Supplementary Table 3 List of samples with discordant concentration categories between LM1010 and UPLC-MS/MS.**
